# Efforts for the Correct Comprehension of Deceitful and Ironic Communicative Intentions in Schizophrenia: A Functional Magnetic Resonance Imaging Study on the Role of the Left Middle Temporal Gyrus

**DOI:** 10.3389/fpsyg.2022.866160

**Published:** 2022-06-14

**Authors:** R. Morese, C. Brasso, M. Stanziano, A. Parola, M. C. Valentini, F. M. Bosco, P. Rocca

**Affiliations:** ^1^Faculty of Communication, Culture and Society, Università della Svizzera italiana, Lugano, Switzerland; ^2^Faculty of Biomedical Sciences, Università della Svizzera italiana, Lugano, Switzerland; ^3^Department of Neuroscience Rita Levi Montalcini, University of Turin, Turin, Italy; ^4^Struttura Complessa di Psichiatria Universitaria, Dipartimento di Neuroscienze e Salute Mentale, Azienda Ospedaliera Universitaria “Città della Salute e della Scienza di Torino”, Turin, Italy; ^5^Neuroradiology Unit, Fondazione IRCCS Istituto Neurologico Carlo Besta, Milan, Italy; ^6^Research Group on Inferential Processes in Social Interaction (GIPSI), Department of Psychology, University of Turin, Turin, Italy; ^7^Struttura Complessa di Neuroradiologia, Dipartimento Diagnostica per Immagini e Radiologia interventistica, Azienda Ospedaliera Universitaria “Città della Salute e della Scienza di Torino”, Turin, Italy

**Keywords:** pragmatic communication, fMRI, schizophrenia, sincere, deceitful, ironic, communicative intentions, left middle temporal gyrus

## Abstract

Deficits in social cognition and more specifically in communication have an important impact on the real-life functioning of people with schizophrenia (SZ). In particular, patients have severe problems in communicative-pragmatics, for example, in correctly inferring the speaker’s communicative intention in everyday conversational interactions. This limit is associated with morphological and functional alteration of the left middle temporal gyrus (L-MTG), a cerebral area involved in various communicative processes, in particular in the distinction of ironic communicative intention from sincere and deceitful ones. We performed an fMRI study on 20 patients with SZ and 20 matched healthy controls (HCs) while performing a pragmatic task testing the comprehension of sincere, deceitful, and ironic communicative intentions. We considered the L-MTG as the region of interest. SZ patients showed difficulties in the correct comprehension of all types of communicative intentions and, when correctly answering to the task, they exhibited a higher activation of the L-MTG, as compared to HC, under all experimental conditions. This greater involvement of the L-MTG in the group of patients could depend on different factors, such as the increasing inferential effort required in correctly understanding the speaker’s communicative intentions, and the higher integrative semantic processes involved in sentence processing. Future studies with a larger sample size and functional connectivity analysis are needed to study deeper the specific role of the L-MTG in pragmatic processes in SZ, also in relation to other brain areas.

## Introduction

Deficits in social cognition and more specifically in communication have an important impact on the real-life functioning of people with schizophrenia (SZ) (Fett et al., 2011; Javed and Charles, 2018; Green et al., 2019). In particular, patients suffering from SZ have shown a wide range of deficits in the communicative-pragmatic domain, characterized by a severe impairment of the comprehension of the speaker’s communicative intention in everyday conversational interactions (Brüne and Bodenstein, 2005; Mazza et al., 2008; Colle et al., 2013; Bambini et al., 2016; Bosco and Parola, 2017; Bosco et al., 2019; Parola et al., 2018, 2021a,2021b; Pawełczyk et al., 2018). Communicative-pragmatics, i.e., the ability to use language to convey meaning in a specific context (Levinson, 1983), is linked to the Theory of Mind (ToM), i.e., the ability to attribute mental states to oneself and others, however, these two abilities do not completely overlap (Bambini et al., 2016; Bosco et al., 2018, 2019; Parola et al., 2018). Rocca et al. (2016) showed that the clustering of patients according to their understanding of lies and complex sarcasm overlap well with their real-life functioning. Colle et al. (2013) showed that patients with SZ, compared to healthy controls (HC), have lower accuracy in the correct comprehension of sincere, deceitful, and ironic communicative acts.

Previous functional magnetic resonance imaging (fMRI) studies investigated brain activations during irony comprehension tasks in people with SZ (Rapp et al., 2013; Varga et al., 2013) and, by comparing the neural activations of patients and HC, revealed reduced activations in different cortical areas of the right hemisphere. In detail, a diminished BOLD signal was found in the middle temporal gyrus (MTG), rolandic operculum, post-central gyrus (Rapp et al., 2013), inferior parietal lobule (IPL), middle frontal gyrus (MFG), and temporal pole (Varga et al., 2013). A recent study proposed a pragmatic fMRI process on healthy subjects that simultaneously assessed the understanding of non-sense, literal or sincere, deceptive, and ironic communicative acts (Bosco et al., 2017). The authors found an activation of the left middle temporal gyrus (L-MTG) for the correct understanding of ironic communicative intentions when compared with the sincere and deceitful communicative acts. In particular, the activation of this cortical region was stronger under the ironic conditions as it was observed from the contrast between the correct understanding of ironic and deceptive communicative acts. The authors concluded that the L-MTG could play a pivotal role in the understanding of more complex communicative acts, like the ironic ones, that require demanding inferential steps. Several former neuroimaging studies on the samples of healthy populations demonstrated the activation of this cortical area in many language-related processes, from semantic integration (Noppeney and Price, 2004) to speech processing (Booth et al., 2002; Bitan et al., 2007; Hickok and Poeppel, 2007; Binder et al., 2009; Holle et al., 2010; Binder and Desai, 2011; Branco et al., 2018). A meta-analysis by Ferstl et al. (2008), for example, indicated that the L-MTG plays a key role in the comprehension and analysis of coherence of a text. Moreover, in subjects with SZ, a reduction of its gray matter (GM) volume was found in people with the first stage of schizophrenia (Kuroki et al., 2006; Hu et al., 2013; Guo et al., 2014), in healthy unaffected siblings of people with SZ (Hu et al., 2013; Guo et al., 2014), and in patients with a long duration of illness (Onitsuka et al., 2004). This finding was also confirmed by a large meta-analysis on GM alteration in SZ (van Erp et al., 2018). Moreover, a reduced thickness of this cortical area (Cui et al., 2018) and an altered resting-state functional connectivity with the left inferior frontal gyrus in the language network (Zhang et al., 2017) correlated with the presence of verbal hallucination. The functional connectivity of the L-MTG was also reduced during a specific process of gesture and speech integration. In particular, the reduced coactivation of this cortical area with the left superior temporal gyrus was associated with attention deficits and concretism in the proverb interpretation (Wroblewski et al., 2020). Furthermore, alterations of the functional connectivity of the L-MTG were reported in a meta-analysis on formal thought disorders (Cavelti et al., 2018). Reduced connectivity of the dorsal posterior part of this area was linked to a deficit in semantic discrimination, ToM, and social cognition (Wensing et al., 2017). Finally, alterations of the L-MTG connectivity were also found at a structural level in terms of increased fractional anisotropy and an augmented radial diffusivity in the white matter tract connecting the L-MTG with the ventral posterior cingulate cortex (Joo et al., 2018).

Considering the role played by the L-MTG in high–level inferential processes and the structural and functional alteration of this cortical region in patients with SZ, we performed an fMRI study on the comprehension of sincere, deceitful, and ironic communicative intentions in this clinical population. Indeed, the comprehension of both pragmatic phenomena requires to recognize the conflict between the literal meaning of a sentence and the speaker’s private mental states (Bosco and Bucciarelli, 2008; Parola and Bosco, 2022), and it is associated with the activation of a cerebral network extending to the frontotemporal and frontoparietal areas (Harada et al., 2009; Bohrn et al., 2012; Spotorno et al., 2012; Uchiyama et al., 2012; Bosco et al., 2017; Filik et al., 2019). At the same time, previous studies have also shown that the comprehension of irony, compared to comprehension of deceit, can activate additional areas, in particular the L-MTG, since understanding irony may require more complex inferential processes necessary to recognize that the content of an ironic utterance also contrasts with the knowledge the speaker shares with the partner (Bosco et al., 2017).

Furthermore, in clinical samples of people suffering from SZ, differences in the correct understanding of deceitful and ironic communicative intentions were shown; patients performed worse in the linguistic and extralinguistic understanding of ironic communicative acts compared to deceitful ones (Colle et al., 2013; Parola et al., 2018). In addition, when assessing error patterns in understanding sincere, deceptive, and ironic communicative acts, patients with SZ, compared to HC, showed a bias in favor of deception, i.e., they tended to misclassify sincere and ironic communicative acts by attributing to them a deceptive communicative intention (Parola et al., 2021a).

The aim of this study was to investigate the neural correlations related to the correct understanding of sincere, deceitful, and ironic intentions in people with SZ, focusing, in particular, on the role of the L-MTG. The choice to compare the same speech act proffered with an ironic vs. deceitful intention is motivated by two reasons; the first is that no fMRI study has previously compared these two pragmatic phenomena in subjects with SZ and the second is to verify whether, even within a clinical sample, differential activation of the L-MTG discriminates against the correct understanding of ironic vs. deceitful communicative intentions. We expected that patients with SZ, as compared to HC, will perform worse in the comprehension of deceitful and ironic communicative acts while, at a neural level, the L-MTG will be differently activated in the clinical group as compared to HC.

## Materials and Methods

### Participants

Individuals diagnosed with schizophrenia (*n* = 20) according to DSM-5 criteria (American Psychiatric Association, 2013) and healthy comparison individuals (*n* = 20) matched with average age, sex, and education were included in this study (Table 1). All participants gave their informed consent and took part in the study voluntarily. The study was approved by the Local Research Ethics Committee (protocol number: 0076364). All participants met the following inclusion criteria: (1) be aged between 18 and 65 years, (2) be right-handers, (3) have no history of neurological illness, and (4) demonstrate basic cognitive and linguistic abilities by achieving a cutoff score in the following neuropsychological tests, Test di Intelligenza Breve (TIB, Colombo et al., 2002), the Italian equivalent of the National Adult Reading Test (NART; Nelson and Willison, 1991; cutoff score 70) and two sub-scales (Comprehension of written words and comprehension of written sentences) of the Aachener Aphasie Test (AAT, Huber et al., 1983; cutoff score 112/120), and (5) be Italian native speakers. People diagnosed with SZ also met the following criteria: (1) have no other mental disorder and (2) be clinically stable, i.e., absence of hospitalization and treatment modification in the last 6 months. Finally, healthy controls (HC) met the following inclusion criteria: (1) no current use of psychoactive drugs and (2) no personal and familiar history of psychiatric disorders.

**TABLE 1:** Demographic characteristics.

	SZ group (*n* = 20)	HC group (*n* = 20)	Statistic (*F*/χ^2^)	*p*-value
Age (years)	41,5 (11,3)	42,1 (10,8)	0.003	0.954
Gender (M/F)	13/7	13/7	0.000	1
Education (years)	13,7 (4,3)	13,85 (4,2)	0.012	0.912

*SZ, schizophrenia; HC, healthy controls; M, male; F, female. Continuous variables are expressed as means and standard deviations (SD).*

### Psychiatric Assessment

The Positive and Negative Syndrome Scale (PANSS, Kay et al., 1987) was used to assess symptom severity. The dimensions “disorganization” and “positive symptoms” were calculated as proposed by Wallwork et al. (2012). Negative symptoms were rated with the Italian version of the Brief Negative Symptoms Scale (BNSS, Mucci et al., 2015) and grouped into the factors “avolition,” consisting of anhedonia, asociality, and avolition, and “expressive deficit,” including the blunted affect and alogia (Kirkpatrick et al., 2011; Strauss et al., 2012). Depressive symptoms were evaluated with the Calgary Depression Scale for Schizophrenia (CDSS, Addington et al., 1993). Functioning was evaluated with the Personal and Social Performance Scale (PSP, Morosini et al., 2000). Antipsychotic dosage was converted to chlorpromazine (CPZ) equivalent dose using the conversion methodology proposed by Leucht et al. (2016). Extrapyramidal symptoms were rated with the Simpson Angus Scale (SAS, Simpson and Angus, 1970).

### Task and Functional Magnetic Resonance Imaging

#### Experimental Material

To test participants’ comprehension of sincere, deceitful, and ironic communicative acts we used an experimental study consisting of 36 short written stories, each of them composed of two parts, i.e., a context scenario and a target sentence. The scenario described the context in which the target sentence was realized. In each context, two characters have a brief communicative interaction and the target sentence represents the final part of their short dialogue (see Supplementary Section 1.1 for an example of the stories). We used three different context scenarios to represent three communicative intentions, namely, deceitful, ironic, and sincere (control condition). Context scenarios and target sentences were comparable to each other in terms of syllables, difficulty, and the number of words (Gulpease readability index–see Supplementary Table 1). The experimental material has already been validated in a previous study (see Bosco et al., 2017 for further methodological details).

#### Experimental Procedure

After the signing of the informed consent by each participant, we described, to each of them, the process that would be performed during the fMRI session. Participants also carried out training to familiarize themselves with the experimental task presented *via* a head coil-mounted display system (Resonance Technology, Inc.Los Angeles, California, United States) using the visual stimuli using the E-Prime software (Psychology Software Tools, Inc., Pittsburgh, PA, United States).

In line with the study of Bosco et al. (2017), each trial of the fMRI experimental study was displayed on the screen in the following order: (i) context- scenario (15 s); (ii) fixation cross (“+”) (5–7 s); (iii) target sentence (6 s); (iv) fixation cross (“+”) (5–7 s); (v) response (4 s); (vi) fixation cross (“+”) (10–12 s) (see Supplementary Figure 1). During the response phase of the fMRI process [as described above “response (4 s)”], each participant was asked to identify the communicative intention expressed during the target sentence and to indicate it by choosing between sincere, deceptive, and ironic options presented on the screen. The indication of the choice was made by pushing a button corresponding to the option presented on the screen.

#### Statistical Analysis of Socio-Demographic and Behavioral Data

Statistical analyses of socio-demographic and behavioral data were performed using the software Statistical Package for Social Sciences, SPSS, version 25.0 for Windows (SPSS, Chicago, IL, United States).

Demographic characteristics and cognitive assessment variables of SZ and HC groups were compared using one-way analysis of variance (ANOVA) for continuous variables and the chi-square test for categorical variables.

Behavioral results, i.e., correct responses during the fMRI process, were analyzed using a two-way repeated-measure analysis of variance (RM-ANOVA). The within-subject factor had three repeated measures (i.e., sincere, deceitful, and ironic) to evaluate whether participants’ accuracy differed between different experimental conditions. The group (SZ or CT) was selected as the between-subject factor.

#### Magnetic Resonance Imaging Data Acquisition

The MRI data were collected using a 3.0 T MRI Scanner (Philips Ingenia) using a 32-channel array head coil, provided with Philips specific eyeglasses (Resonance Technology, Inc.). MRI data acquisition was carried out at the Neuroimaging Center (Centro di Neuroimmagini–CNI) of the Neuroscience Institute of Turin (NIT) of the University of Turin, located in the Azienda Ospedaliera Universitaria “Città della Salute e della Scienza di Torino” in Turin, Italy. MRI acquisition parameters of a previous study (Bosco et al., 2017) were applied. In detail, Echo-Planar Image sequence (EPI) with TR/TE = 3,000/30 ms, 32 slices, matrix size = 96 × 96, slice gap = 0.5 mm, field of view (FOV) = 224 × 224 mm^2^, and flip angle = 90°, with slices aligned on the AC-PC line, consisting of 230 volumes, was used during two runs for collecting functional images. Structural images were recorded applying a T1-weighted sequence (TR 8.1 ms, TI 900 ms, TE 3.7 ms, voxel size 1 × 1 × 1 mmł).

#### Functional Magnetic Resonance Imaging Data Analysis

The fMRI data were analyzed using SPM12 (Wellcome Department of Cognitive Neurology, London, United Kingdom) in Matlab (Mathworks, Cherborn, MA, United States). We applied the same analysis procedure as that used in a previous study (Bosco et al., 2017). In particular, for the preprocessing analysis of each participant, functional images were initially realigned spatially and then co-registered to their mean. They, subsequently, were normalized to the MNI (Montreal Neurological Institute) space and then smoothed applying an 8 mm Gaussian Kernel.

After preprocessing analysis, to investigate the participants’ correct comprehension of the communicative intention compared to the HC, we convolved the onset times related to the target sentences with the canonical hemodynamic response function (HRF) using a General Linear Model (GLM) (Friston et al., 1995).

At the first level, we calculated, for each participant, three separate regressors of interest considering only correct responses, one for each experimental condition, sincere, deceitful, and ironic.

At the second level, we applied a full factorial design aiming at investigating the neural difference between SZ patients and HC comprehension of the communicative intentions. We employed a two-way RM-ANOVA with the within-subject factor, communicative intention, at three levels, i.e., sincere, deceitful, and ironic. At first, for an exploratory purpose, we applied a whole-brain analysis at *p* < 0.001 uncorrected thresholds. In this exploratory analysis, we checked whether the fMRI results of HC were in line with a previous study (Bosco et al., 2017) by performing the following contrasts within the HC group, deceitful condition vs. sincere condition and ironic condition vs. sincere condition. Within the SZ group, all possible contrasts among the three experimental conditions (i.e., sincere, deceitful, and ironic) were carried out.

Finally, the following between-group contrasts, Family-Wise Error (FEW) cluster-level corrected (*p* < 0.05), were examined, i.e., [SZ group (deceitful condition vs. sincere condition)] vs. [HC group (deceitful condition vs. sincere condition)] and [SZ group (ironic condition vs. sincere condition)] vs. [HC group (ironic condition vs. sincere condition)].

Then, given our hypothesis about the role of the L-MTG, a small volume correction applying a sphere of 10 mm radius centered on coordinates from our previous study (*x* = −49; *y* = −37; *z* = −2; Bosco et al., 2017) was used. In this analysis, a between-group contrast was analyzed for each condition (i.e., sincere, deceitful, and ironic) to compare the recruitment of the L-MTG in the correct comprehension of communicative intentions between patients with SZ and HC.

In addition, separately for the SZ group and HC group, correlations with task performance and with clinical and cognitive variables were evaluated using multiple regression analysis with Statistical Parametric Mapping 12.

## Results

### Demographic and Clinical Characteristics

Demographic characteristics of SZ and HC groups were homogeneous (Table 1).

Clinical characteristics of the SZ group are shown in Table 2. The age at onset and the duration of illness were quite heterogeneous with a mean of 27.5 and 14.4 years, respectively. Scores of positive symptoms and disorganization dimensions were globally low, with an average of 7.45 (min 4–max 28) and 6.25 (min 3–max 21) reciprocally. Higher scores were observed in the avolition dimension (mean score 21.85; min 0–max 42) and to a lesser extent in the expressive deficit factor (mean score 11.60; min 0–max 30). Depressive symptoms were globally low or mild (mean 4.1; min 0–max 18). The personal and social functioning was partly reduced with a mean of the PSP score of 61.6 (min 0–max 100). A total of 18 subjects with SZ were treated with an atypical antipsychotic drug with an in-label daily dosage. Two patients were taking a typical antipsychotic drug. None of them was using clozapine. Extrapyramidal side effects were negligible (mean SAS = 0.75).

**TABLE 2:** Clinical characteristics.

Age at Illness Onset, Years	27.50 (7.99)
Duration of Illness, Years	14.60 (9.81)
PANSS positive, Score	7.45 (1.82)
PANSS disorganization, Score	6.25 (2.63)
BNSS avolition, Score	21.85 (9.22)
BNSS expressive deficit, Score	11.65 (7.40)
CDSS, Total score	4.10 (5.08)
PSP, Score	61.60 (13.05)
CPZ equivalent, mg/day	371.80 (144.87)
SAS, Total score	0.75 (2.15)

*PANSS, Positive and Negative Syndrome Scale; BNSS, Brief Negative Symptoms; CDSS, Calgary Depression Scale for Schizophrenia; PSP, Personal and Social Performance Scale; CPZ, chlorpromazine; SAS, Simpson Angus Scale.*

*Data are shown as means and standard deviations (SD).*

### Behavioral Results

**FIGURE 1:**
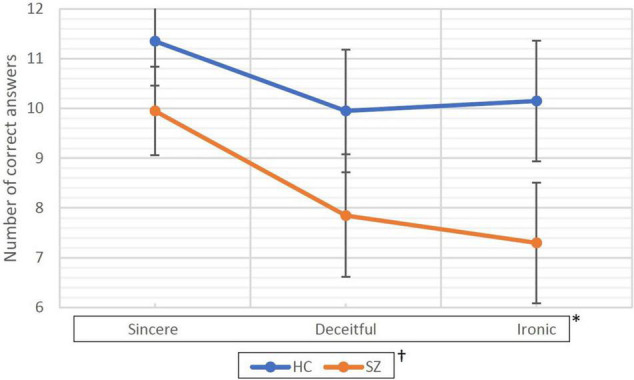
Correct responses collected during the fMRI task. HC, healthy controls; SZ, patients with schizophrenia. Data are represented as the mean of correct answers (dots) and 95% confidence intervals (bars). * and † represent statistical significance of the two factors of the ANOVA for repeated measure. * represents within-subjects factor that are experimental conditions (i.e., sincere, deceitful, and ironic communicative intentions to be understood) chosen as repeated measure. The *p* associated with this factor was <0.001. † represents the between-subjects factor (group factor, HC vs. SZ) and was associated with a *p* = 0.001.

The mean rate (95% CI) of correct responses obtained during the process is shown in Table 3 and Figure 1. In the HC group, the mean rate was 11.35/12 (94.6%) under the sincere condition, 9.95/12 (82.9%) under the deceitful condition, and 10.15/12 (84.6%) under the ironic one. Global accuracy was 31.45/36 (87.4%). The SZ group showed a poorer performance with a mean rate of correct answers of 9.95/12 (82.9%), 7.85/12 (65.4%), and 7.30/12 (60.3%) under the sincere, deceitful, and ironic conditions, respectively. Global accuracy was 25.10/36 (69.7%). The Mauchly’s sphericity test performed for the two-way RM-ANOVA was not significant (*W* = 0.84; *p* = 0.058), therefore, we followed the sphericity assumption. The within-subject factor under sincere, deceitful, and ironic experimental conditions upon repeated measures and the between-subject factor (group HC vs. SZ) were both highly significant with *F* = 12,503 (*p* < 0.001) and *F* = 12,096 (*p* = 0.001), respectively. The interaction between the two factors, i.e., experimental condition × group, was not significant with *F* = 1.451 (*p* = 0.241).

**TABLE 3:** Correct responses in the fMRI task.

	HC	SZ
Sincere	11.35 (10.45–12.24)	9.95 (9.06–10.84)
Deceitful	9.95 (8.72–11.17)	7.85 (6.62–9.08)
Ironic	10.15 (8.94–11.36)	7.30 (6.09–8.51)

*HC, healthy controls; SZ, patients with schizophrenia.*

*Data are shown as means and 95% confidence intervals (95% CI).*

### Functional Magnetic Resonance Imaging Results

**FIGURE 2:**
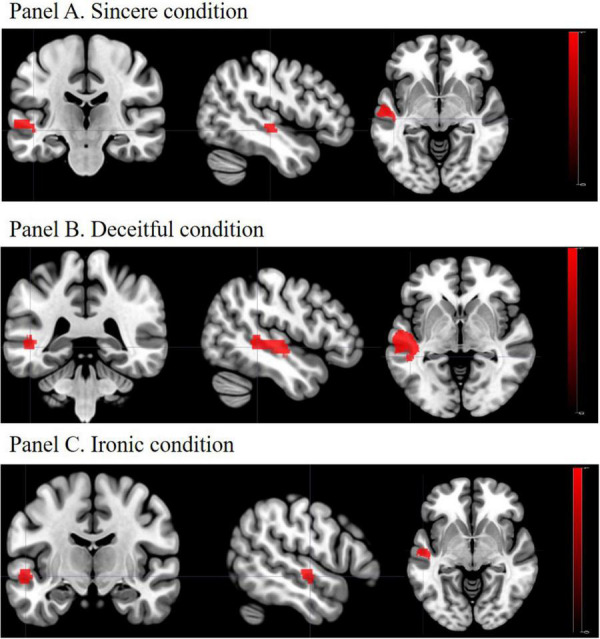
Activation of the L-MTG: SZ vs. HC. **Panel A**. Sincere condition; **Panel B**. Deceitful condition; **Panel C**. Ironic condition. L-MTG, left middle temporal gyrus; SZ, schizophrenia group; HC, healthy controls group. In all the experimental conditions patients with SZ showed higher activation of the L-MTG, as compared to HC, when correctly comprehended the communicative intention proposed in the task.

Between-group linear contrasts revealed significant stronger activations in the L-MTG in the SZ group vs. the HC group under all the experimental conditions (Table 4 and Figure 2). Within-group linear contrasts are shown in Supplementary Table 2 and Supplementary Figures 2, 3. In the linear contrasts performed within the HC group, we found activations in the left dorsolateral prefrontal cortex (*x* = −42; *y* = 18; *z* = 29), in the left inferior frontal gyrus (*x* = −51; *y* = 27; *z* = 5), and in the left middle frontal gyrus (*x* = −50; *y* = 14; *z* = 37) under the deceitful condition when compared to the sincere one (control condition). Under the ironic condition, as compared with the sincere one, activations were found in the left middle frontal gyrus (*x* = −53; *y* = 18; *z* = 29), in the left dorsolateral prefrontal cortex (*x* = −45; *y* = 10; *z* = 32), in the left inferior frontal gyrus (*x* = −57; *y* = 25; *z* = 8), and in the left middle temporal gyrus (*x* = −52; *y* = −37; *z* = 4). Within the SZ group, none of the contrasts performed showed differences between the sincere, deceptive, and ironic conditions. Between-group (SZ vs. HC) whole brain contrasts showed greater activation in the group of patients exclusively in the L-MTG under the ironic condition (Supplementary Table 3). No suprathreshold voxels (not statistically significant), emerged from the correlation between L-MTG activation and task performance and clinical and cognitive variables in the SZ and HC groups.

**TABLE 4:** Functional magnetic resonance imaging results; activation of the L-MTG; SZ vs. HC.

Experimental condition	MNI coordinates			*Z*-score	*p* (FWE-corr.)
	*X*	*Y*	*Z*		
*Sincere* L-MTG	−53	−20	−2	3.07	0.048
*Deceitfu*l L-MTG	−62	−30	2	3.21	0.033
*Ironic* L-MTG	−51	−23	5	3.18	0.044

*Activation of the L-MTG for the linear contrasts: (i) sincere SZ group vs. sincere HC group, (ii) deceitful SZ group vs. deceitful HC group, and (iii) ironic SZ group vs. ironic HC group.*

*L-MTG, left middle temporal gyrus; SZ, schizophrenia group; HC, healthy controls group; MNI, Montreal Neurological Institute; FEW-corr., family-wise error correction. Peak activity coordinates are given in MNI space.*

*Linear contrasts were computed using a small volume correction (SVC) with a sphere of 10 mm with a statistical threshold of p < 0.05 family-wise error corrected for multiple comparisons.*

## Discussion

The aim of the study was to investigate the neural correlations related to the correct understanding of sincere, deceitful, and ironic intentions in people with SZ, focusing, in particular, on the role of the L-MTG. The contrasts between the SZ group and the HC group (SZ vs. HC) showed a higher recruitment of the L-MTG under all the experimental conditions. This phenomenon was also found at a whole-brain level in the comprehension of ironic communicative intentions. These results demonstrate a higher involvement of this cerebral area in patients with SZ when they answered correctly during the process.

Previous fMRI studies on healthy subjects have demonstrated that the activation of the L-MTG during communicative pragmatic tasks has been associated with the recognition of ironic (Eviatar and Just, 2006) and sarcastic statements (Uchiyama et al., 2006), with efforts in the comprehension of linguistic tasks (Bohrn et al., 2012; Rapp et al., 2013), with the pragmatic inferential ability (Jang et al., 2013), and with the integration of information from different semantic systems (Davey et al., 2016). In patients with SZ, this cerebral area showed a reduction of the GM volume (Onitsuka et al., 2004; Kuroki et al., 2006; Hu et al., 2013; Guo et al., 2014; van Erp et al., 2018) and altered functional connectivity with other cerebral areas in the language network and in language-related fMRI processes (Wensing et al., 2017; Zhang et al., 2017; Joo et al., 2018; Wroblewski et al., 2020). According to this evidence, the L-MTG would play a key role in pragmatic communication processes and would be deficient in subjects with SZ. We assume that patients suffering from SZ could need a greater inferential effort and a stronger L-MTG activation to be able to understand correctly all the communicative acts, including the sincere ones. In other words, this stronger activation might be a form of over-compensation required by the patients to answer the questions of the fMRI process correctly. Moreover, as revealed by the whole-brain between-group comparison, this effort would be even more evident for the understanding of irony, which may require more complex inferential processes. However, since the L-MTG has been found to be involved in various linguistic processes, such as semantic integration of word meaning (Vandenberghe et al., 2002; Noppeney and Price, 2004), in the comprehension of texts and analysis of text coherence (Ferstl et al., 2008), and in controlling the retrieval of semantic information (Davey et al., 2016), the activation found in our results might be ascribed to some extent to other cognitive and linguistic processes involved in the correct understanding of communicative intentions.

Focusing on the within-group liner contrasts, fMRI results in the HC group partially replicated those of Bosco et al. (2017; see Supplementary Results: Supplementary Table 2 and Supplementary Figures 2, 3). In detail, the following discrepancies emerged: in the deception vs. sincerity contrast, a greater activation of the right cerebellum was not detected, and in the irony vs. sincerity comparison, we did not observe a greater involvement of the left temporoparietal junction and of the right cerebellum. These inconsistencies in the cerebral hemodynamic response to the process (i.e., the BOLD signal) could be derived from the different demographic characteristics of the samples of healthy subjects in the two experiments, a homogeneous group of university students in Bosco et al. (2017), and a more heterogeneous group of participants (range 23–60 years old), which matched with patients with SZ in this study. Following this explanation, as reported by D’Esposito et al. (2003), higher age can negatively affect the BOLD signal, thus reducing the number of areas emerging from the linear contrasts. Despite these differences, within the HC group, the communicative pragmatic process was associated with the activation of brain areas partially overlapping with those described by Bosco et al. (2017), hence demonstrating a good degree of replicability of the experimental results.

Unexpectedly, within the group of patients with SZ, there was no difference between the experimental control condition (sincere) and the deceitful and ironic conditions. As previously proposed for the higher activation of L-MTG in the clinical group under all the experimental conditions, this phenomenon might be explained by the marked inferential effort made by patients to correctly understand communicative intentions regardless of the experimental conditions. Following this explanation, all experimental conditions, including the baseline (i.e., sincere), would facilitate a marked brain activation such as to “saturate” the BOLD signal. In other words, this putative high level of activation, regardless of the experimental conditions, makes it impossible to distinguish the brain areas specifically activated in the correct understanding of deceptive and ironic communicative intentions within the SZ group as also under the baseline condition, the same brain areas were strongly activated. However, this is only one possible interpretation of the result obtained. In fact, since no differences emerged between the three experimental conditions, what was observed could be the result of non-specific phenomena unrelated to the understanding of communicative intentions.

At a behavioral level, patients showed more difficulty than HC under all three experimental conditions, especially in irony understanding. This result is in accordance with previous pragmatic studies carried out on subjects with SZ (Brüne and Bodenstein, 2005; Mazza et al., 2008; Colle et al., 2013; Bambini et al., 2016; Bosco et al., 2019; Parola et al., 2021b). Moreover, we found that both patients and HC demonstrated greater difficulties in the correct comprehension of deceptive and ironic experimental conditions compared to the sincere one. This trend is coherent with the results of the previous study on healthy subjects (Bosco et al., 2017).

The impossibility to perform correlation analysis between the activation of L-MGT during the process and behavioral, clinical, and cognitive variables is probably due to the relatively small sample size. In fact, the relatively low number of subjects for each group (*n* = 20 for both SZ and HC groups) did not allow one to find any statistically significant voxel within the L-MTG, a prerequisite to carry out the correlation analysis.

The main limitation of this study is the relatively small sample that conditioned the types of fMRI analyses carried out. Analyses on multiple regions of interest belonging to the language network and analyses on functional connectivity of the L-MTG during the task and the resting state are needed to better understand the role of this specific cortical area in pragmatic abilities. With a similar aim, DTI analyses on the connectivity of WM of the L-MTG in relation to the performance in the task proposed might also be performed. These types of studies can verify the activity of L-MTG in relation to other cerebral areas and possibly bring out, also within the group of subjects with SZ, differences between the three different experimental conditions, but require a larger sample size.

Despite these limitations, this study has two important strengths. The first one is related to the fMRI task; for the first time. in a clinical sample of patients with SZ, the neural activation was investigated during the comprehension of both deceitful and ironic speech acts. In fact, in previous fMRI studies in people with SZ, only irony, and not deceit, comprehension, was investigated (Rapp et al., 2013; Varga et al., 2013). The second concerns the selection criteria of the sample as only clinically stable, right-handed, without marked cognitive deficits, and non-bilingual native Italian speaker patients were enrolled. This homogeneity of the sample has reduced possible confounding factors by limiting misleading results. Similarly, the choice of analyzing images related solely to the correct answers eliminated possible brain activations due to the misinterpretation of the communication intentions.

In conclusion, this study adds a small piece to the panorama of fMRI studies on pragmatics in schizophrenia characterizing the role of a brain area involved in the understanding of complex communicative acts, i.e., the L-MTG that is frequently altered for the structure and connectivity in subjects affected by the disorder. However, due to the poor generalizability of the results linked to the low sample size and the methods, this study needs to be integrated with more experiments using whole-brain techniques that confirm the role of L-MTG in pragmatic communication skills, possibly in response to specific rehabilitation programs, as proposed by Gabbatore et al. (2017).

## Data Availability Statement

Due to the anonymity guaranteed in the informed consent paperwork at the time when data were collected, data cannot be publicly shared, and are controlled by the Comitato Etico Interaziendale of the A.O.U. Città della Salute e della Scienza di Torino. Researchers who wish to request access to these data may contact the corresponding author (CB), claudio.brasso@unito.it.

## Ethics Statement

The studies involving human participants were reviewed and approved by Comitato Etico Interaziendale A.O.U. Città della Salute e della Scienza di Torino - A.O. Ordine Mauriziano - A.S.L. Città di Torino. The patients/participants provided their written informed consent to participate in this study.

## Author Contributions

RM: data collection, data curation, task preparation, fMRI data analysis, and writing–review and editing. CB: data collection, data curation, statistical analysis, methodology, and writing–original draft. MS and MV: data collection and writing–review and editing. AP: data collection, task preparation, and writing–review and editing. FB: conceptualization, methodology, task preparation, project administration, supervision, and writing–review and editing. PR: conceptualization, funding acquisition, project administration, supervision, and writing–review and editing. All authors contributed to the article and approved the submitted version.

## Conflict of Interest

The authors declare that the research was conducted in the absence of any commercial or financial relationships that could be construed as a potential conflict of interest.

## Publisher’s Note

All claims expressed in this article are solely those of the authors and do not necessarily represent those of their affiliated organizations, or those of the publisher, the editors and the reviewers. Any product that may be evaluated in this article, or claim that may be made by its manufacturer, is not guaranteed or endorsed by the publisher.
