# Supplementary material for: Efforts for the Correct Comprehension of Deceitful and Ironic Communicative Intentions in Schizophrenia: A Functional Magnetic Resonance Imaging Study on the Role of the Left Middle Temporal Gyrus
Source: Front Psychol. 2022 Jun 14;13:866160. doi: 10.3389/fpsyg.2022.866160 (PMC9237627; doi:10.3389/fpsyg.2022.866160)
Supplement: Supplementary file 1 [file Data_Sheet_1.docx]

**SUPPLEMENTARY INFORMATION**

**1. Supplementary methods**

1.1. Example of three different context scenarios with a common target sentence

[SINCERE] (S) Tom and Mary decided to go to the mountains the next day. Next morning they wake up and go to the kitchen to have breakfast. Mary asks Tom what the weather is like; he looks out of the window and sees that the sun is shining. Tom replies:

[DECEITFUL] (D) Mark knows that the weather forecast is for rain tomorrow, but he wants to persuade Ann to come with him to the seaside despite the bad weather. Ann tells him that she will only come if it is a sunny day, and she asks Mark what the weather is like. Mark replies:

[IRONIC] (I) Frank and Alice are going on a picnic. They take the picnic basket and get in the car to drive to the countryside. They have just arrived when they hear a loud clap of thunder and feel a few drops of rain. Alice shoots Frank a questioning glance. Frank exclaims:

[TARGET SENTENCE] (T) “It’s a beautiful day!”

**Table S1.** The mean number of words, syllables and the Gulpease index for target sentence and the three different context-scenarios (sincere/literal, deceitful, ironic) associated with each target sentence

| Condition | | Mean Length (SD) - number of words | |  | |  | Mean Length (SD) – number of syllabes | | Mean Gulpease (SD) | |  | |
| --- | --- | --- | --- | --- | --- | --- | --- | --- | --- | --- | --- | --- |
| Sincere/Literal Context |  |  | 44.1 (4.3) | | 91.5 (7.8) | | |  | | 67.8 (5.1) | |  |
| Deceitful Context |  |  | 46.3 (4.0) | | 89.7 (8.4) | | |  | | 71.7 (4.5) | |  |
| Ironic Context |  |  | 44.5 (5.5) | | 91.0 (9.6) | | |  | | 72.2 (.5.9) | |  |
| Target Sentence |  |  | 5.4 (1.8) | | 10.9 (2.2) | | |  | | 94.5 (6.6) | |  |

**Fig. S1 Structure of the experimental task employed in the study**


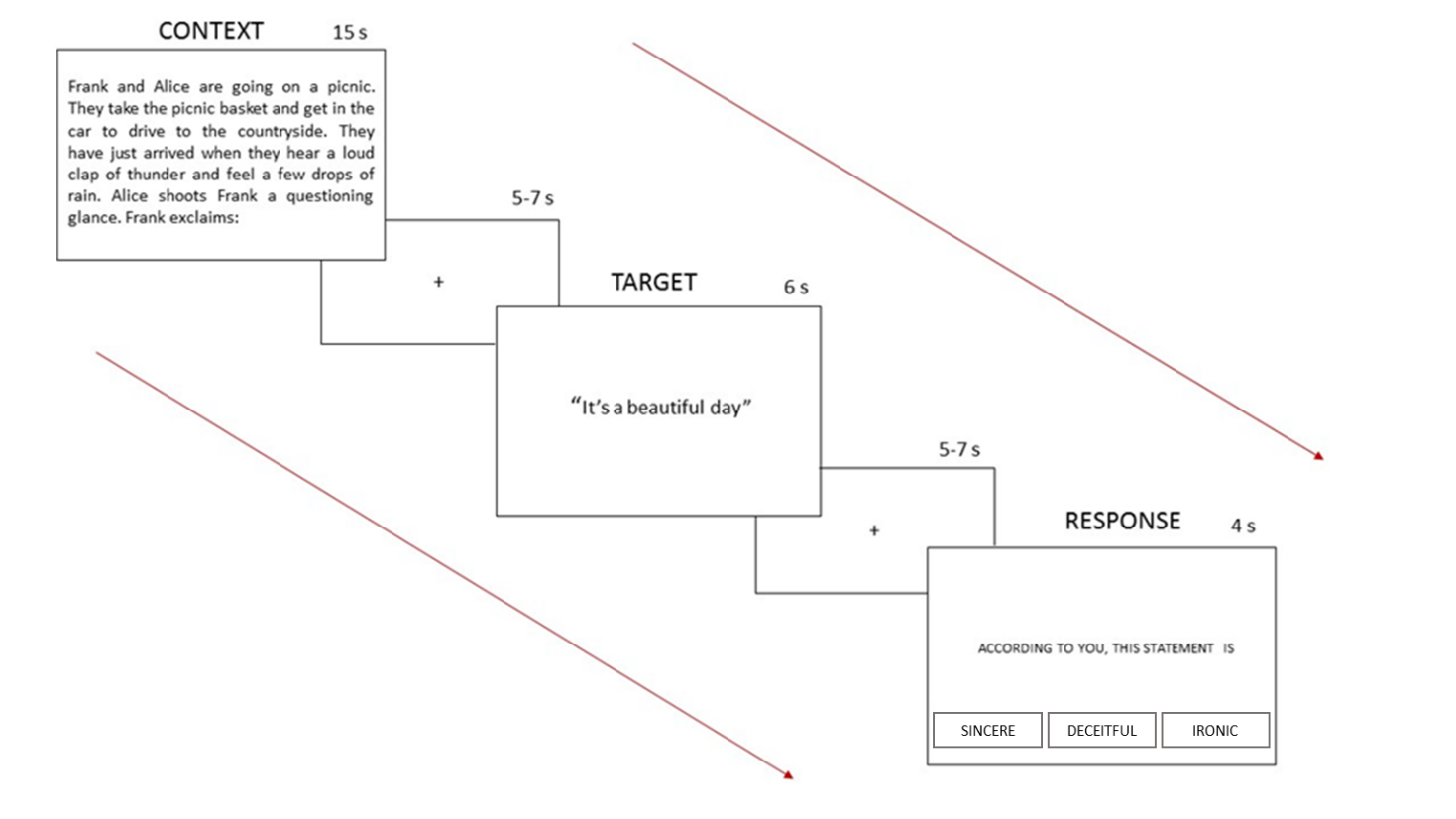
Each trial started with the presentation on the screen of the context story for 15 sec, followed by a fixation cross (“+”) for 5-7 sec; target sentence for 6sec, a fixation mark (“+”) for 5-7 sec and response screen for 4 sec.

**2. Supplementary results**

2.1 Exploratory contrasts within the healthy controls (HC) group

**Figure S2 – Exploratory contrasts within the HC group**

*A. Deceitful condition vs sincere condition B. Ironic condition* vs *sincere condition*

**
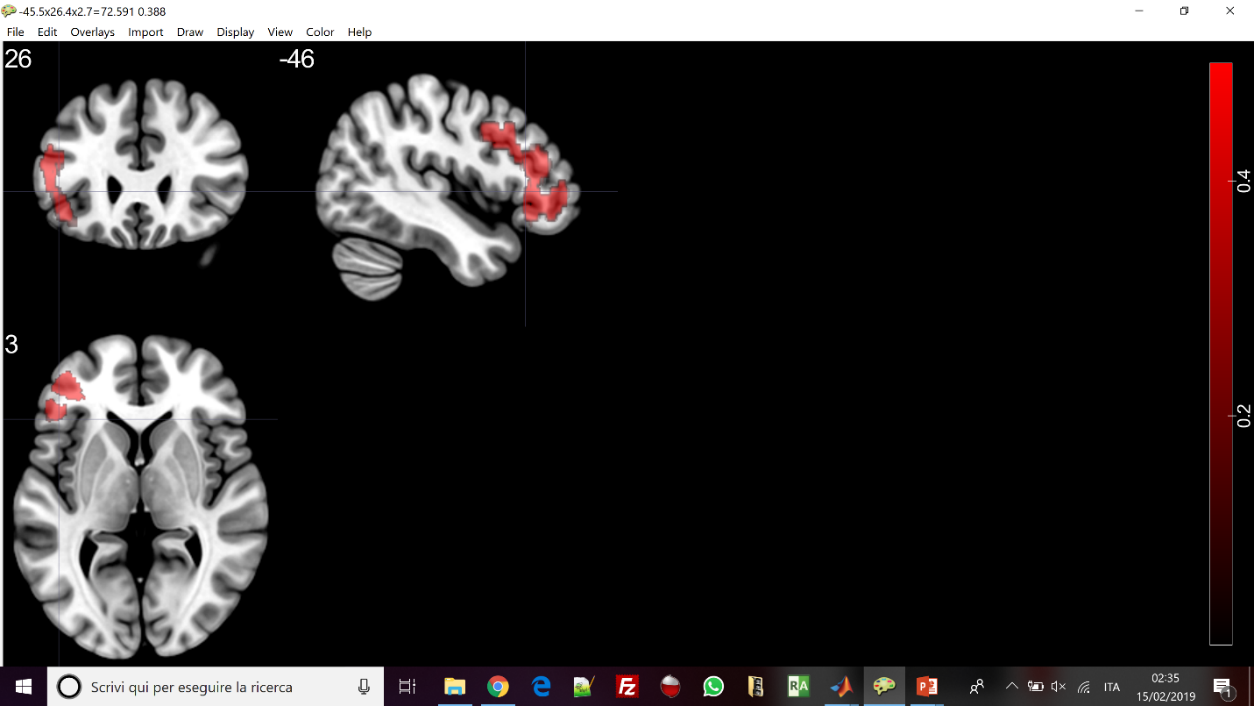
** *
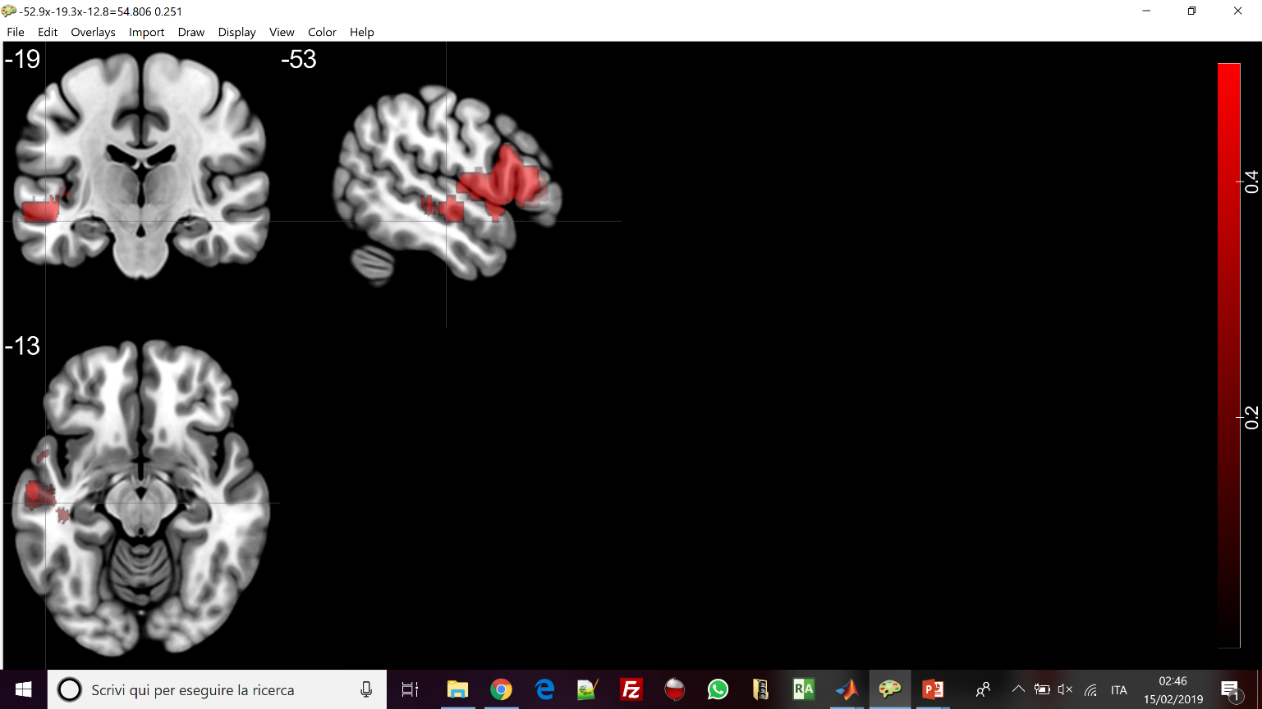
*

HC: healthy controls. Brain activation maps of the contrasts between deceitful and ironic conditions versus sincere (control) condition within the HC group. Cerebral areas involved in the activation are listed in table S2. Whole brain analyses at p < 0.001 uncorrected threshold.

**Table S2 – Exploratory contrasts within the HC group**

Peak activity coordinates are given in MNI space. Whole brain analyses at p < 0.001 uncorrected threshold.

| **Significantly activated brain regions** | | | | |
| --- | --- | --- | --- | --- |
| **Contrasts of interest** | **MNI Coordinates** | | |  |
|  | *X* | *Y* | *Z* |  |
| ***Deceitful condition vs sincere condition*** |  |  |  |  |
| Left dorsolateral prefrontal cortex (L-DLPFC) | -42 | 18 | 29 |  |
| Left inferior frontal gyrus (L-IFG) | -51 | 27 | 5 |  |
| Left middle frontal gyrus (L-MFG) | -50 | 14 | 37 |  |
| ***Ironic condition vs sincere condition*** |  |  |  |  |
| Left middle frontal gyrus (L-MFG) | -53 | 18 | 29 |  |
| Left dorsolateral prefrontal cortex (L-DLPFC) | -45 | 10 | 32 |  |
| Left inferior frontal gyrus (L-IFG) | -57 | 25 | 8 |  |
| Left middle temporal gyrus (L-MTG) | -52 | -37 | 4 |  |

2.2. Exploratory contrasts within the group of patients with schizophrenia (SZ group)

**Figure S3 – Exploratory contrasts within the SZ group**

*A.1. Ironic condition* vs *Sincere condition B.1. Deceitful condition vs Sincere condition*


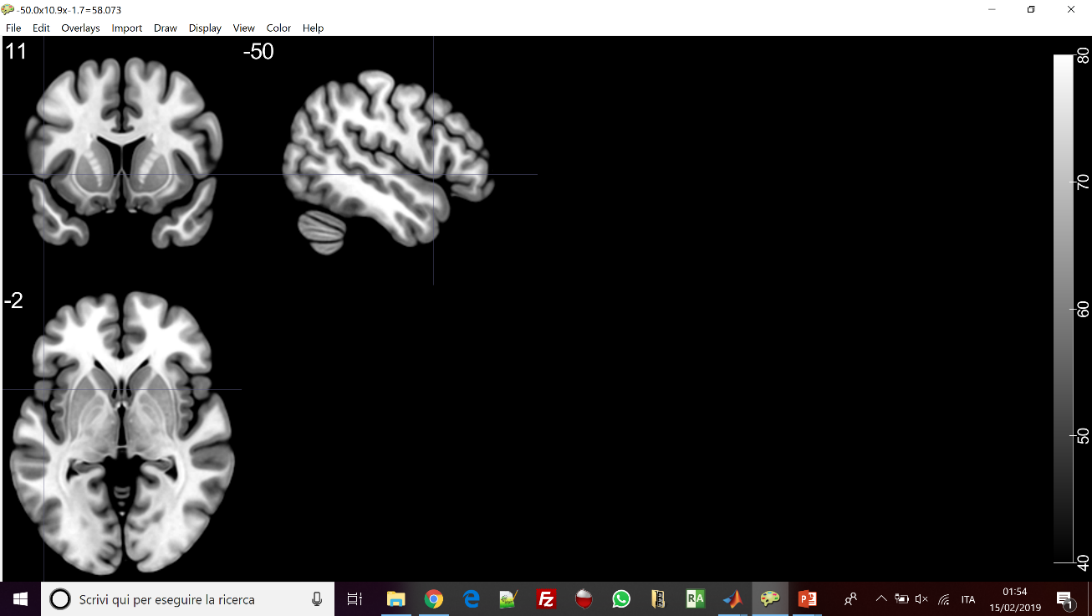

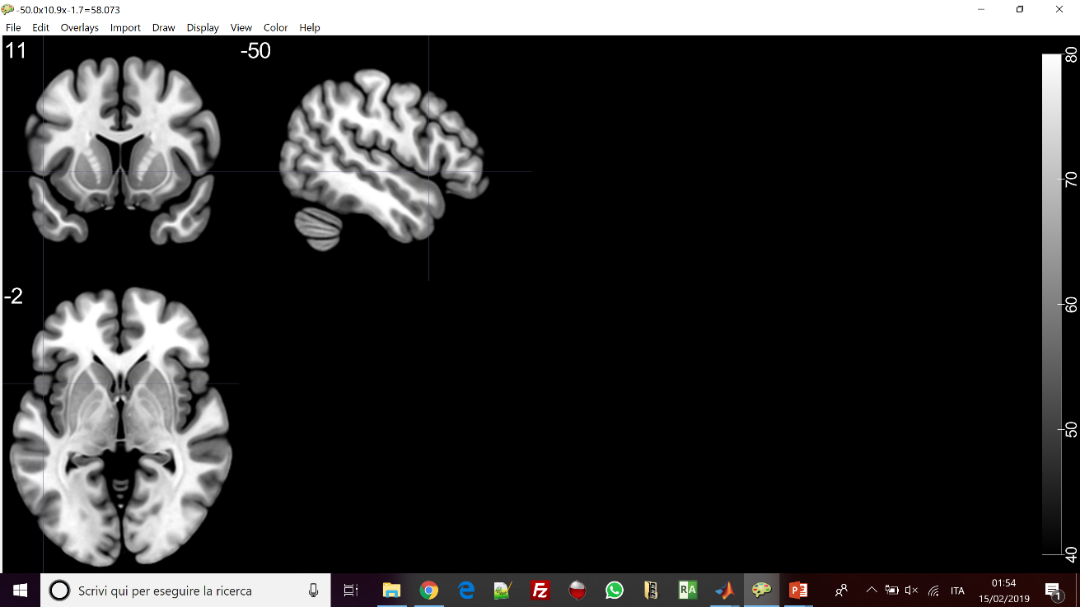


*A.2. Sincere condition* vs *Ironic condition B.2. Sincere condition vs Deceitful condition*


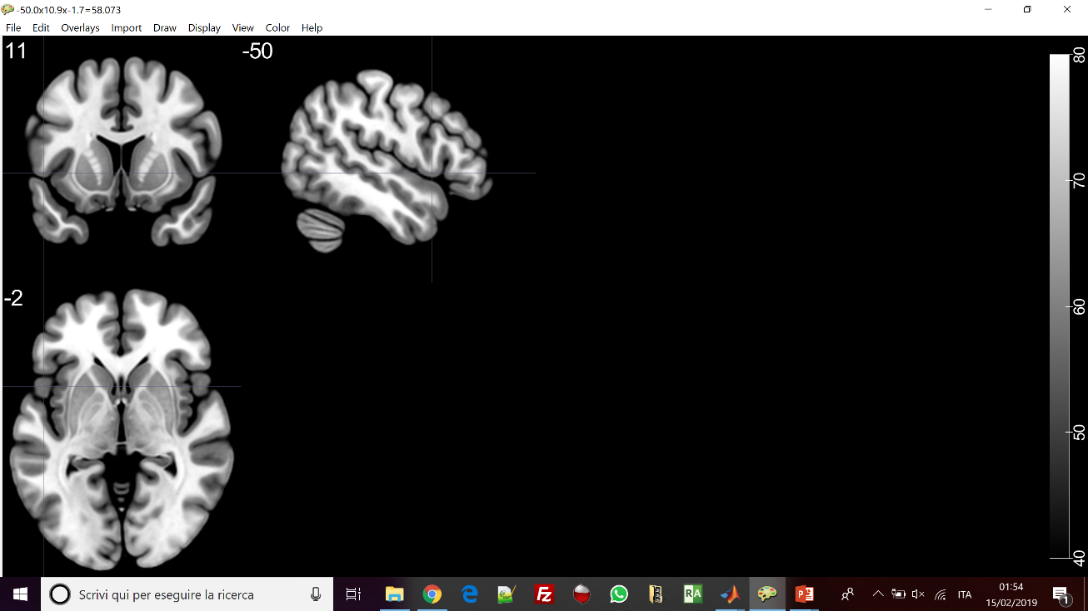

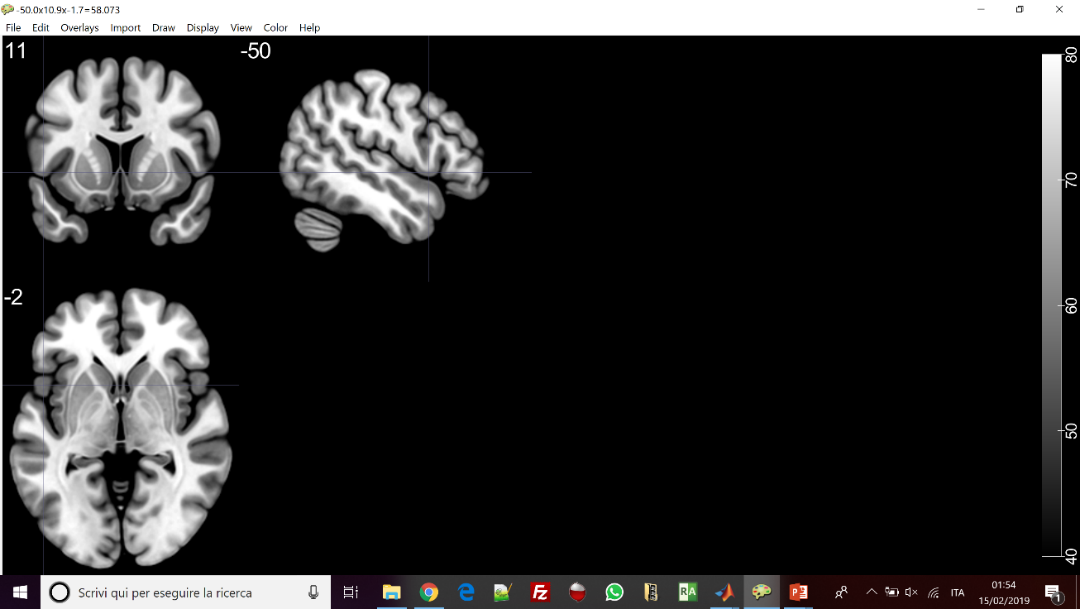


*C.1. Deceitful condition* vs *Ironic condition C.2. Ironic condition vs Deceitful condition*


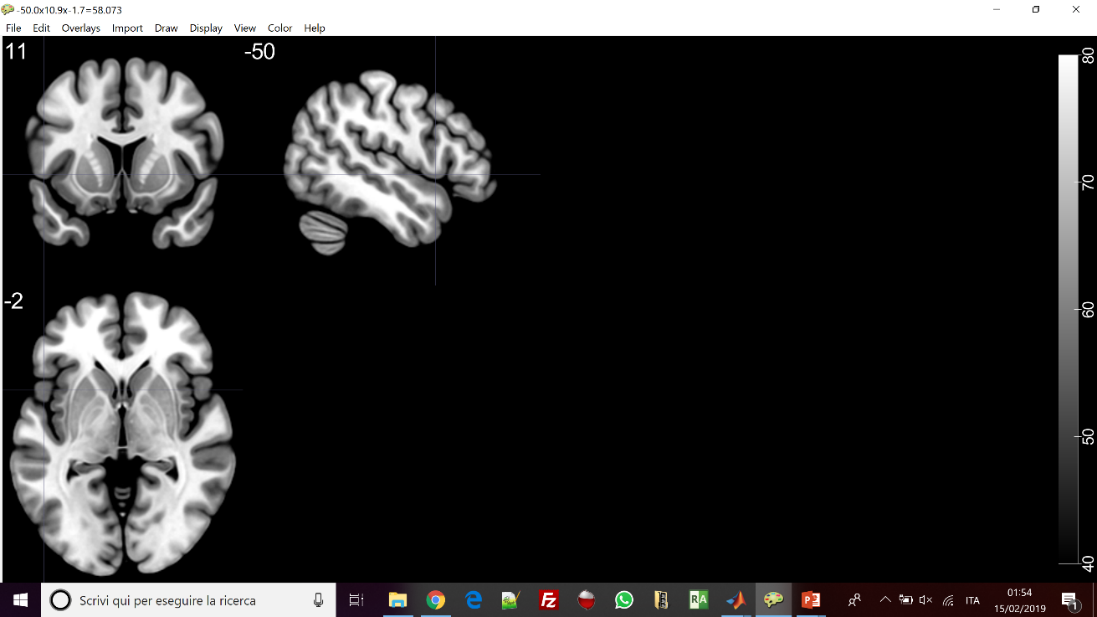

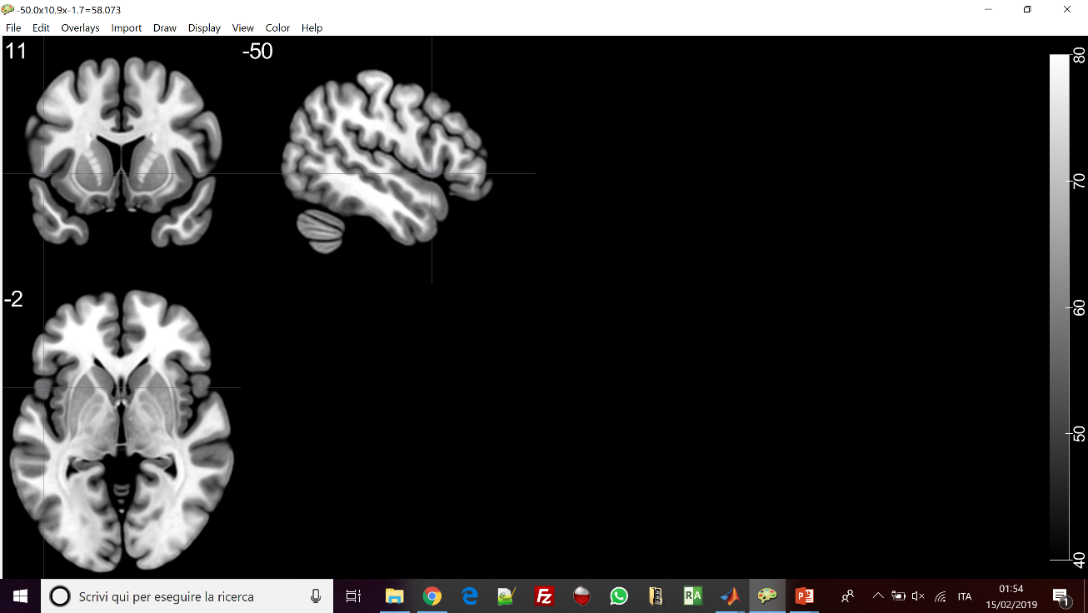


Brain activation maps of all the possible contrasts between the three experimental conditions (i.e.: sincere, deceitful, and ironic) within the SZ group. With a whole brain analysis at p < 0.001 uncorrected threshold, no activation was found.

2.3 Whole brain contrasts between the SZ group vs HC group

**Table S3. Whole brain contrasts between the SZ group vs HC group**

| **Brain regions** | | | | |
| --- | --- | --- | --- | --- |
| **Contrasts of interest** | **MNI Coordinates** | | |  |
|  | *X* | *Y* | *Z* |  |
| ***Ironic condition vs sincere condition*** |  |  |  |  |
| Left middle temporal gyrus (L-MTG) | -48 | -29 | 11 |  |
|  | -47 | -20 | 15 |  |

Whole brain SPM analysis. The table indicates the brain regions involved in the following contrasts between the SZ group vs HC group: deceitful vs sincere, ironic vs sincere (p < 0.05, FWE cluster-level corrected). No activation was found for the contrast deceitful vs sincere.
